# Hesperidin Attenuates Hypothyroidism-Induced Lung Damage in Adult Albino Rats by Modulating Oxidative Stress, Nuclear Factor Kappa-B Pathway, Proliferating Cell Nuclear Antigen and Inflammatory Cytokines

**DOI:** 10.3390/biomedicines11061570

**Published:** 2023-05-29

**Authors:** Walaa Hegazy, Hader I. Sakr, Manal Abdul Hamid, Mohamed A. Abdelaziz, Marwa Salah, Eman S. Abdel Rehiem, Adel Abdel Moneim

**Affiliations:** 1 Histology Division, Basic Science Department, Faculty of Physical Therapy, Nahda University, Beni-Suef 62511, Egypt; 2 Department of Medical Physiology, Faculty of Medicine, Cairo University, Cairo 11562, Egypt; hadersakr@kasralainy.edu.eg; 3 Department of Medical Physiology, Medicine Program, Batterjee Medical College, Jeddah 21442, Saudi Arabia; 4 Cell Biology, Histology and Genetics Division, Zoology Department, Faculty of Science, Beni-Suef University, Salah Salem St., Beni-Suef 62511, Egypt; 5 Basic Medical Sciences Department, College of Medicine, Prince Sattam Bin Abdulaziz University, Al-Kharj 11942, Saudi Arabia; 6 Medical Physiology Department, Faculty of Medicine, Al-Azhar University, Cairo 11651, Egypt; 7 Molecular Physiology Division, Zoology Department, Faculty of Science, Beni-Suef University, Salah Salem St., Beni-Suef 62511, Egypt

**Keywords:** hesperidin, hypothyroidism, lung injury, histopathological, immunohistochemical, inflammatory cytokines, oxidative stress

## Abstract

The occurrence of worsening pulmonary function has been connected to hypothyroidism (HPO). Hesperidin (HES) was suggested to have antioxidant, anti-proliferative, and anti-inflammatory potential. Our study’s objective was to determine whether HES could reduce carbimazole (CBZ)-induced lung injury more effectively than Eltroxin (ELT) in adult male albino rats or not. At random, 32 rats were distributed into four groups: Group I: normal control, to induce HPO, the remaining three groups were given CBZ (20 mg/kg/day) dissolved in distilled water for 1 week. They were then split up into three groups. Group II: orally administered CBZ (20 mg/kg b.w in water/day), Group III: HES (200 mg/kg/day) dissolved in 1% carboxymethyl-cellulose + CBZ treated, and Group IV: ELT (0.045 mg/kg/day) dissolved in distilled water + CBZ treated. All treatments were delivered for 12 weeks. Blood was collected to assess thyroid-stimulating hormone (TSH) and thyroid hormones (THs). Lung injury was evaluated based on the pulmonary content of interleukin (IL)-35, IL-6, and tumor necrosis factor-alpha (TNF-α), along with the estimation of lipid peroxidation, catalase, glutathione levels, superoxide dismutase, heme oxygenase-1 (HO-1), and nuclear factor erythroid 2-related factor 2 (Nrf2). The histological, ultrastructural, and immunohistochemical study of nuclear factor Kappa-B (NF-κB) and inducible nitric oxide synthase (iNOS), together with estimating the proliferation of cells using Antigen Ki-67 in lung tissue were performed. HES and ELT primarily suppressed variable lung damage mechanisms by suppressing TSH, the NF-κB/TNF-α pathway, iNOS, lipid peroxidation, Ki-67, and inflammatory mediators. On the other hand, they improved THs, antioxidant parameters, and the Nrf2/HO-1 pathway. HES and ELT exhibited an ameliorative effect that was reflected in the histopathological, immunohistochemical, and ultrastructural results. These results indicate that HES is a pneumoprotective agent that could be a promising treatment for oxidative stress, inflammation, and proliferation.

## 1. Introduction

Hypothyroidism (HPO) influences the respiratory system and other human organs and systems. Pulmonary functions may decline because of a drop in the thyroid hormones (THs) level in HPO [1]. Carbimazole (CBZ) has been utilized to induce experimental HPO [2]. The active form of CBZ, methimazole (1-methyl-2-mercaptoimidazole), prevents tyrosine iodination stimulated by thyroid peroxidase, resulting in a decrease in circulating THs [3]. Reduced THs were linked to reduced O_2_ usage and CO_2_ production rates [4]. HPO usually has a deceptive beginning that is usually symptomless [5]. It is well known that HPO reduces ATP production and mitochondrial oxygen consumption [6]. HPO can impact pulmonary functioning, resulting in a restrictive pattern of lung damage. As a result, the rate of breathing decreases, which results in a reduction in pulmonary ventilation [7]. HPO can induce many respiratory symptoms, ranging from mild dyspnea to the respiratory system failing completely [8]. Lung volumes that are affected by how quickly air flows out of the lung (dynamic lung functions) are significantly reduced in HPO [9].

Because the lungs have a huge surface area continuously in touch with atmospheric oxygen and contaminants, it is a primary site of reactive oxygen species (ROS) [10]. Malondialdehyde (MDA) levels were incremented in subclinical HPO because HPO is linked to upgraded oxidative stress [11]. HPO provides evidence that THs can modulate the antioxidant systems [12]. Particularly, more prone to the harmful effects of oxidants is alveolar Type II pneumocyte [13]. HPO induces deviations in metabolic and immune processes [14]. Chronic inflammation per se is considered a source of oxidative stress, that heightens the inflammatory response through nuclear transcription factors activation that are redox sensitive [15]. The activation of inflammatory cells, which release inflammatory mediators to alter the behavior of other cell types, is the fundamental mechanism of inflammation [16]. HPO boosted the production of cytokines and oxidative stress, which disrupted cell signaling and life. Numerous investigations suggested that HPO patients have altered serum interleukins (ILS) [17,18]. Both lung inflammation and bronchoconstriction are considered the most common indicator of lung damage [19]. By affecting phospholipids, surfactants, and increased vessel permeability, cytokines led to acute lung injury [20]. Experimental HPO can cause fibrosis by increasing the expression of the collagen Type I gene [21]. Hypoxia could be the cause of HPO-related lung fibrosis [22].

Eltroxin (ELT) has long been the primary treatment for HPO and is one of the most commonly prescribed medications worldwide. ELT is an artificial TH used to treat HPO. ELT upregulates thyroid-stimulating hormone (TSH) receptors, causing increased T4 and triiodothyronine (T3) production. When T3 moves in the nucleus, it binds to the nuclear TH receptor (TR) very tightly. The hormone-receptor complex then interacts with the retinoid X receptor (RXR) forming TR/RXR heterodimer complex that in turn interacts with the thyroid hormone response element (TRE) sequences in the target genes’ promoter regions regulating its expression. To obtain the greatest benefit for patients, ELT should be utilized wisely [23]. Treatment of subclinical HPO with ELT may postpone the progression of HPO overtly and alleviate neuropsychiatric symptoms, musculoskeletal pain, and coronary artery disease danger [24,25].

Flavonoids are phenolic natural chemicals in vegetables, fruits, and plant roots that function as antioxidants in biological systems. Flavonoids have antioxidant, anti-inflammatory, anti-bacterial, anti-mutagenic, and anti-cancer properties [26,27]. The nuclear factor Kappa-B (NF-κB) aggravates the inflammatory insult generating significant inflammatory alterations in a variety of important organs, particularly the lungs [28]. Flavonoids are compounds that have the capacity to disrupt the NF-κB signaling pathway engaged in regulating the inflammatory enzymes and cytokines involving interleukin (IL)-6 and tumor necrosis factor-alpha (TNF-α) [29]. Recent works have shown that natural medicine such as flavonoid active ingredients can decrease inflammatory and oxidative stress and have protective effects against lung injury through the erythroid 2 related factor 2 (Nrf2) signaling pathway [30,31]. Activated Nrf2 is an intracellular antioxidant protective signal distributed in several organs, including the lungs, where antioxidation and detoxification cascades are routinely processed [32]. Stress-inducible protein heme oxygenase-1 (HO-1) is important for oxidative stress protection and lung inflammation management [33]. Abundant natural flavonoids can activate Nrf2, which is effective for treating inflammatory and oxidative stress-related diseases, involving acute lung damage [34].

Satsuma mandarin, sweet orange, bitter orange, and lemon are examples of citrus fruits that contain hesperidin (HES), a flavanone glycoside that can be extracted in considerable concentrations and is easily accessible [35]. According to Bayomy et al. [36], HES has antioxidative, anti-inflammatory, and anticarcinogenic properties. HES is a three-hydroxylated molecule with a higher antioxidant efficacy stimulating cellular antioxidant-inhibiting enzymes compared to other flavanones [37]. Experiments on male and female rats revealed that HES is nontoxic, quickly digested, has no accumulative effects, and causes no allergic reactions [38].

The putative effect of HES on HPO-induced lung damage has received very little attention. The objective of our study was to ascertain the influence of CBZ-prompted HPO on morphofunctional and biochemical changes in adult male albino rat lungs, as well as the prospective protective effect of HES on these changes and the possible roles of NF-κB, Nrf2, inducible nitric oxide synthase (iNOS), and HO-1.

## 2. Materials and Methods

### 2.1. Chemicals and Kits

Chemical Industries Development (El Haram, Giza, Egypt) provided the CBZ (5 mg in tablet form) which does not store above 25 °C. ELT (50 mcg tablets) was obtained from GlaxoSmithKline GmbH (Prinzregentenplatz, München, Bayern Germany) and it was kept cool, dry, and protected from light, at below 25 °C temperature. HES powder (Sigma Chemical Co., St. Louis, MO, United States of America (USA)) was stored away from direct sunlight at 2–4 °C. El Gomhoria, a chemical and medical trading company in Egypt, provided the carboxymethyl-cellulose (vehicle of HES). Other compounds with high analytical purity were purchased from common commercial sources. The free triiodothyronine (FT3), free thyroxine (FT4), and TSH kits for quantitative determination were provided by Siemens Healthcare Diagnostics (Emil-von-Behring-Strasse 76 35041 Marburg, Germany), and these tests were carried out in compliance with the manufacturer’s methodology.

### 2.2. Animals

32 male albino rats in good health, 130–150 g in weight were supplied by the Nahda University animal house. Male adult albino white rats (*Rattus norvegicus*) were kept under standard conditions in properly-ventilated plastic containers of 12 h dark/light cycle, temperature (22 °C ± 5 °C), and humidity (55% ± 5%), rice straw bedding, and fed regular laboratory food under the same ambient conditions. Rats were anesthetized with light anesthetic inhalation of diethyl ether (5%) and sacrificed by decapitation.

They were allowed 14 days before the experimental period to become accustomed to the laboratory environment. The Institutional Animal Care and Use Committee at Beni-Suef University approved the experiment (BSU-IACUC, No. 020-123).

### 2.3. Experimental Protocol

At random, the animals were separated into four equal groups (n = 8). Rats in the control group were not disturbed or subjected to any stress. They were given 0.5 mL of distilled water orally daily for 90 days. To induce HPO, the three further groups were given CBZ (20 mg/kg/day) [39] dissolved in distilled water for 1 week. They were then split up into three groups. Rats in Group II (CBZ) were given only CBZ as described. In addition to CBZ, rats in Group III (HES + CBZ) received HES (200 mg/kg/day) [40] dissolved in 1% carboxymethyl-cellulose (CMC), and Group IV (ELT + CBZ) received ELT (0.045 mg/kg/day) [41]: dissolved in distilled water. Oral gavage was used for all treatments. Decapitation of all rats which were sacrificed when the experimental time has ended, after 90 days, anesthesia with light diethyl ether inhalation.
**Groups****Experimental Design**I (normal control)Given 0.5 mL distilled water orally daily for 90 daysII (CBZ)Given 0.5 mL CBZ (20 mg/kg/day) orally for 90 daysIII ((HES + CBZ)Given 0.5 mL of CBZ (20 mg/kg/day) dissolved in distilled water followed by HES (200 mg/kg/day) dissolved in 1% CMC for 90 daysIV (ELT + CBZ)Given 0.5 mL CBZ (20 mg/kg/day) dissolved in distilled water followed by ELT (0.045 mg/kg/day) dissolved in distilled water orally for 90 days

### 2.4. Blood Sample Collection and Biochemical Estimations

Blood was drawn from each rat’s retroorbital plexus in non-heparinized tubes and permitted to coagulate. The sera were then separated by centrifugation at 3000 rpm for 15 min at 4 °C and stored at 20 °C in sterilized tubes for THs and TSH analysis.

### 2.5. Analysis of Lung Homogenates

Fresh lungs were collected and fixed, and each rat’s chest was opened to rapidly remove the lung tissues and washed utilizing cold saline. A Teflon homogenizer was used to homogenize the first part of the left lung in cold phosphate-buffered saline (10% *w*/*v*) (Glas-Col, Terre Haute, IN, USA). Clear supernatant was then kept at 20 °C after centrifugation of the homogenates at 3000 rpm for 10 min at 4 °C. ELISA assays kits manufactured by MyBioSource, LLC., (Ontario, ON, Canada) were used depending on the manufacturer’s instructions for the determination of oxidative parameters such as MDA levels, antioxidant parameters such as catalase (CAT), reduction in glutathione (GSH) levels, and superoxide dismutase (SOD) activity in addition to inflammation, which was determined by measurement of IL-6, IL-35, and TNF-α.

### 2.6. Western Blot Assays

Detection of NRF2 and HO-1 by Western Blot technique (usingV3 Western Workflow™ Complete System, Bio-Rad^®^, Hercules, CA, USA): In short, we used an ice-cold radio-immunoprecipitation assay (RIPA) buffer with phosphatase and protease inhibitors supplement (50 mmol/L sodium vanadate, 0.5 mM phenylmethylsulphonyl fluoride, 2 mg/mL aprotinin, and 0.5 mg/mL leupeptin) to extract proteins from tissue homogenates. This was followed by 20 min centrifugation at 12,000 rpm. Bradford assay was used to determine protein concentration in each sample. We separated equal amounts of protein (25 ± 5 µg of total protein) by a Bio-Rad Mini-Protein II system using SDS/polyacrylamide gel (10%) electrophoresis. The protein was transferred to polyvinylidene difluoride membranes (Pierce, Rockford, IL, USA) with a Bio-Rad Trans-Blot system. Then, the membranes were washed and blocked for an hour with 5% (*w*/*v*) skimmed milk powder in PBS at room temperature. After the blocking, the blots were developed using antibodies for NRF2, HO-1, and β-actin provided by (Thermo Scientific, Rockford, IL, USA; with dilution 1:1000) incubated overnight at 4 °C with gentle shaking at pH 7.6. Following washing, secondary antibodies labeled with peroxidase (Rockland Immunochemicals, Gilbertsville, PA, USA; with dilution 1:3000) were added, and the membranes were incubated for an hour at 37 °C. Band intensity was analyzed using the ChemiDoc imaging system with Image Lab software version 5.1 (Bio-Rad Laboratories Inc., Hercules, CA, USA). After normalization for β-actin protein expression, results were expressed as arbitrary units [42].

### 2.7. Histopathological and Immunohistochemical Analysis

For histological processing, the first part of the right lungs was rapidly fixed for 24 h in 10% neutral buffered formalin. Samples used were prepared as usual, encased in Wax paraffin, sectioned by a microtome at 4–5 μm, and stained by Hematoxylin and eosin (H&E) before examination under an Olympus microscope [43]. Other sections were immunohistochemically stained after being placed on positively charged slides to detect iNOS, NF-κB, and Ki-67 (rabbit polyclonal antibodies) at a dilution of 1:100 (Thermo Scientific/Lab Vision Corporation, Fermont, CA, USA). Hematoxylin was used to stain the sections after they were rinsed with distilled water. As a negative control, phosphate-buffered saline was substituted for the stated primary antibody [44].

### 2.8. Evaluation of Histomorphometry

Image J software was used to do histomorphometric analysis (Leica Qwin 500 image system, Cambridge, UK) [45] to evaluate histopathological lesions in H&E-stained sections. The mean area percentage of inflammatory mononuclear cells, bronchiole width in micrometers (µm), and thickening of interalveolar septa (µm) were assessed. The mean area % for iNOS, NF-kB, and Ki-67 immunoexpression intensity was evaluated. Each rat was evaluated by selecting 10 non-overlapping fields with 400×, a total magnification of 400× at random. The data were represented as means ± SEM (*n* = 6)

### 2.9. Ultrastructural Evaluation

Next, 1 mm^3^ of the second part of the right lung was sliced, fixed in 3% glutaraldehyde, rinsed in phosphate buffer, and post-fixed for one hour in isotonic 1% osmium tetroxide at 4 °C (pH 7.4). The preparation of sections for electron microscopic investigation was carried out following the method of Bozzola and Russell [46]. Toluidine blue was used to stain semi-thin sections (1 μm), while ultrathin sections (80 ± 10 nm) were created using ultra-microtome glass knives. Uranyl acetate and lead citrate were used to stain the samples, and a transmission electron microscope was used for examination (Joel CX 100).

### 2.10. Statistical Analysis

Statistical Package for the Social Sciences (SPSS) for Windows version 20.0 (SPSS, Chicago, IL, USA) was utilized to analyze the data. The mean and standard error of the results were calculated (M ± SE). Comparisons were examined using a One Way Analysis of Variance. Duncan’s multiple range test was used to see the significant differences (*p* < 0.05) between the groups. Shapiro-Wilk test was applied for all groups and approved that the investigated data is normally distributed, therefore, the One Way ANOVA test is applied.

## 3. Results

### 3.1. Biochemical Results

Significant decrease in FT3, FT4, and IL-35 in group II compared to the control group. FT3 and IL-35 in the hypothyroid Group treated with HES or ELT were significantly higher than those in group II. ELT did not increase significantly FT4 in Group IV compared to Group II, and HES significantly increased FT4 in Group III compared to the CBZ-treated group. Meanwhile, there were significant increases in TSH, IL-6, and TNF- α comparing Group II to the control group. TSH values in the hypothyroid group treated with HES or ELT were similar to the control group, furthermore, HES and ELT significantly reduced IL-6 and TNF- levels when compared to the normal control group (Table 1).

**Figure 1:**
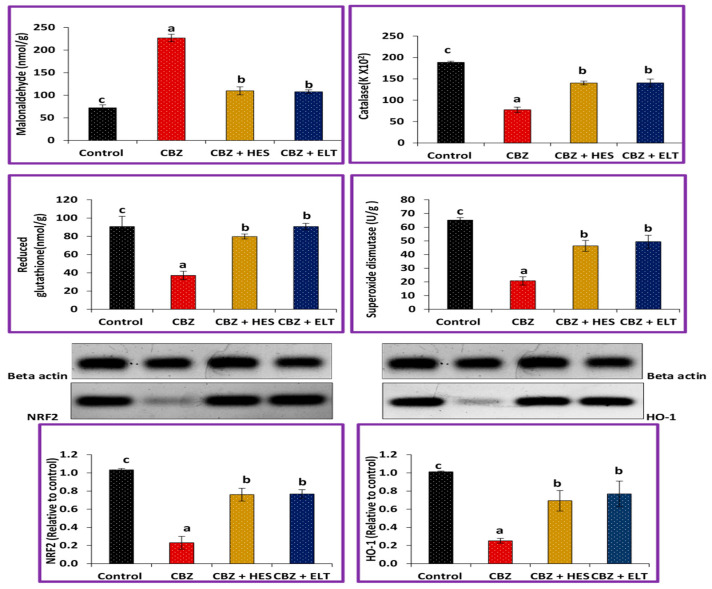
The potential preventive impact of HES and ELT against CBZ-caused alterations in malondialdehyde (MDA) levels, catalase (CAT), reduced glutathione (GSH) levels, superoxide dismutase (SOD) activities, signaling pathway of nuclear factor erythroid 2-related factor 2/heme oxygenase 1 (Nrf2/HO-1) of all experimental groups. This test was repeated two times. Values with different superscript letters are considered significantly different (*p* < 0.05). a: there is a significant difference compared with the control at *p* < 0.05. b, c: there is a significant difference compared with the CBZ group at *p* < 0.05.

The MDA level was significantly increased in Group II compared to the control (Group I). Both HES and ELT in groups III and IV, respectively, showed a significantly decreased MDA production. Moreover, CAT, GSH, SOD, Nrf2, and HO-1 were significantly reduced in Group II compared to the control. Group II’s results for CAT, GSH, SOD, Nrf2, and HO-1 were significantly decreased compared to Groups III and IV, (Figure 1).

### 3.2. Histological Changes and Morphometric Findings

**Figure 2:**
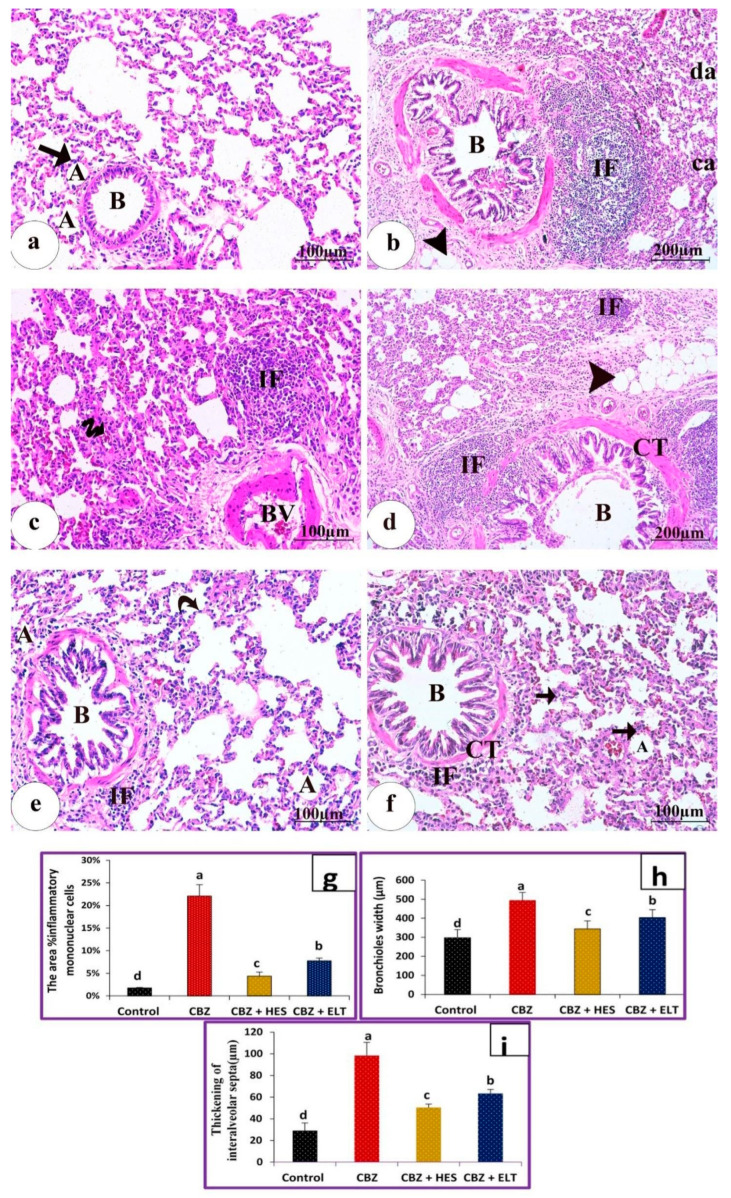
Photomicrographs of lung tissues sections stained with H&E. (**a**) control group showing a normal arrangement of lung tissue, with the typical appearance of bronchioalveolar unit parenchyma, clear alveoli (A) which are lined with type I and II pneumocytes, bronchiole (B), and thin interalveolar septa (arrow). Scale bare = 100 µm. (**b**–**d**) Rat treated with CBZ: demonstrating (**b**) marked hyperplasia of a dilated bronchiole (B), collapsed alveoli (Ca), and other compensatory (da). Thickening of interalveolar septa, severe inflammatory mononuclear cells (IF) in peribronchial and perivascular areas. The bronchiolar walls are moderately thickened and fat cells (arrowhead) are seen. Scale bare = 100 µm. (**c**)) Increased hyperchromatic nuclei number in the cells of the alveolar wall, alveolar hemorrhage (zigzag arrow), vascular thrombosis, and cellular architecture alterations, involving dilatation of congested blood vessels (BV) and much increase of inflammatory mononuclear cells (IF). Scale bare = 100 µm. (**d**) Thickening of alveolar septa, augmentation of inflammatory mononuclear cells (IF) in peribronchial and perivascular areas, and thickening of interstitial connective tissue (CT). Notice dilation of a destructed bronchiole (B) and the appearance of fat cells (arrowhead) was also observed. Scale bare = 200 µm. (**e**) The group treated with HES plus CBZ showing nearly normal, the parenchyma of the bronchioalveolar unit has a typical appearance with clear alveoli. (A) lined with type I and II pneumocyte containing uniform nuclei, bronchiole (B), thin intra alveolar septa (curved arrow), and few infiltrations of inflammatory cells (IF). Scale bare = 100 µm. (**f**) The group treated with ELT plus CBZ showing alveoli (A), mild thickened interalveolar septa (arrows), bronchiole (B), and mild focal thickening of interalveolar interstitial connective tissue (CT) with a few infiltrations with inflammatory cells (IF). Scale bare = 100 µm. (**g**) The mean area % of inflammatory mononuclear cells was revealed in all different studied groups. (**h**) Bronchioles width (µm) was measured in all groups. (**i**) Thickening of interalveolar septa (µm) was measured in all groups. (**g**–**i**). Sample number (*n*) = 6. Groups with the same superscript letter do not differ significantly. a: There is a significant difference compared with the control at *p* < 0.05. b, c, d: there is a significant difference compared with the CBZ group at *p* < 0.05.

The control group showed a normal arrangement of lung tissue, with a typical appearance of bronchioalveolar unit parenchyma, clear alveoli lined with Type I and II pneumocytes, bronchioles, and thin interalveolar septa (Figure 2a). Rats treated with CBZ demonstrated marked hyperplasia of a dilated bronchiole, collapsed alveoli, and other compensatory effects. Thickening of interalveolar septa, significant augmentation of the inflammatory mononuclear cells in peribronchial and perivascular areas, and a moderate thickening of the bronchial walls were also found. Augmentation in the number of hyperchromatic nuclei in the cells of the alveolar wall, alveolar hemorrhage, and significant cellular architectural alterations involving dilatation of congested blood vessels and the appearance of fat cells were also noted (Figure 2b–d). Group III (HES + CBZ) showed a nearly normal appearance of the broncho-alveolar unit parenchyma, with clear alveoli lined with Type I and II pneumocytes containing normal nuclei, bronchioles, thin intra-alveolar septa, and few infiltrations of inflammatory cells (Figure 2e). Group IV (ELT + CBZ) showed nearly clear alveoli thickened interalveolar septa, normal appearance of bronchioles, and mild focal thickening of interstitial connective tissue with a few infiltrations of inflammatory cells (Figure 2f). The mean area percentage of inflammatory mononuclear cells was increased in group II compared to the three other groups. Group II significantly increased inflammatory mononuclear cells, bronchiole width, and the interalveolar septa’s thickness. It is noteworthy that compared to Group II, HES in Group III and ELT in Group IV significantly reduced inflammatory mononuclear cells, bronchiole width, and the interalveolar septa’s thickness. HES in Group III demonstrated a potent downregulation of mononuclear cell trafficking, bronchiole width, and the interalveolar septa’s thickness that was significantly greater than ELT’s capacity (Figure 2i).

### 3.3. Immunohistochemical Results

**Figure 3:**
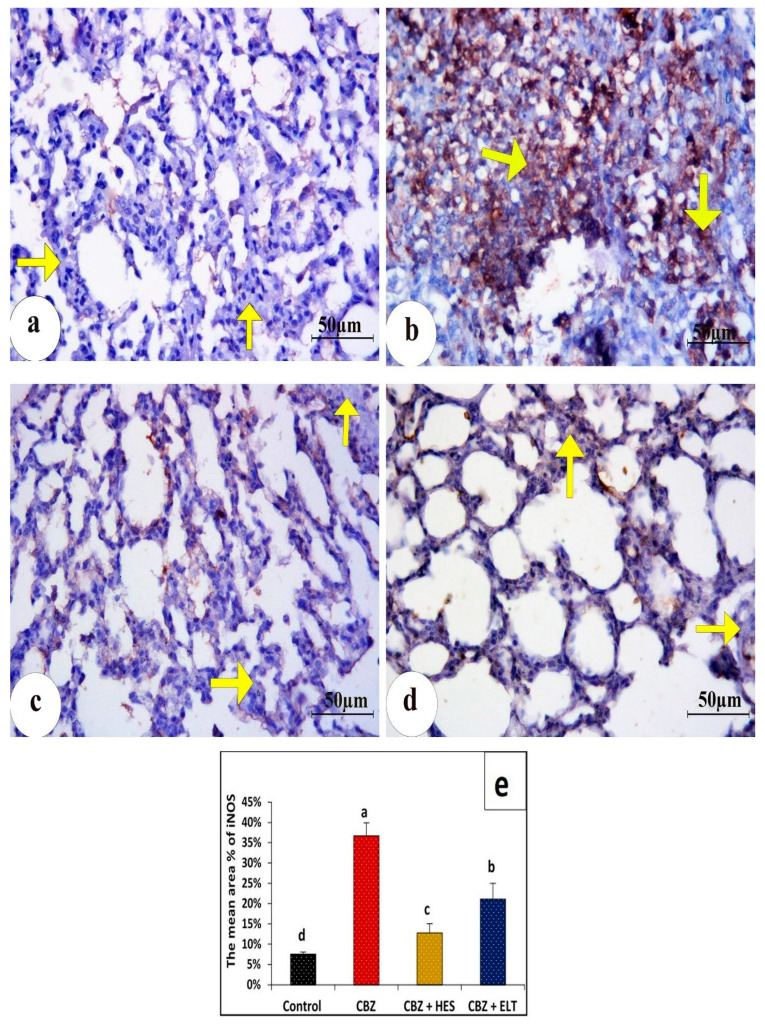
Photomicrographs of immunohistochemical expression of iNOS in lung tissue. (**a**) normal control rats with very few immunohistochemical expressions of iNOS (arrows). (**b**) Rats treated with only CBZ showing a marked augmentation of the brown color representing the positive immunostaining (arrows). (**c**) animals treated with HES plus CBZ revealing a small degree of immunostaining (arrows). (**d**) Sections from rats treated with ELT plus CBZ unveiled moderate immunohistochemical expression (arrows). (iNOS immunostaining, ×400). (**e**) Bar charts representation of the statistical analysis of the comparative quantification of the immunohistochemical expression for iNOS in lung tissues of all groups. Groups with the same superscript letter do not differ significantly. Sample number (*n*) = 6. a: there is a significant difference compared with the control at *p* < 0.05. b, c, d: there is a significant difference compared with the CBZ group at *p* < 0.05.

**Figure 4:**
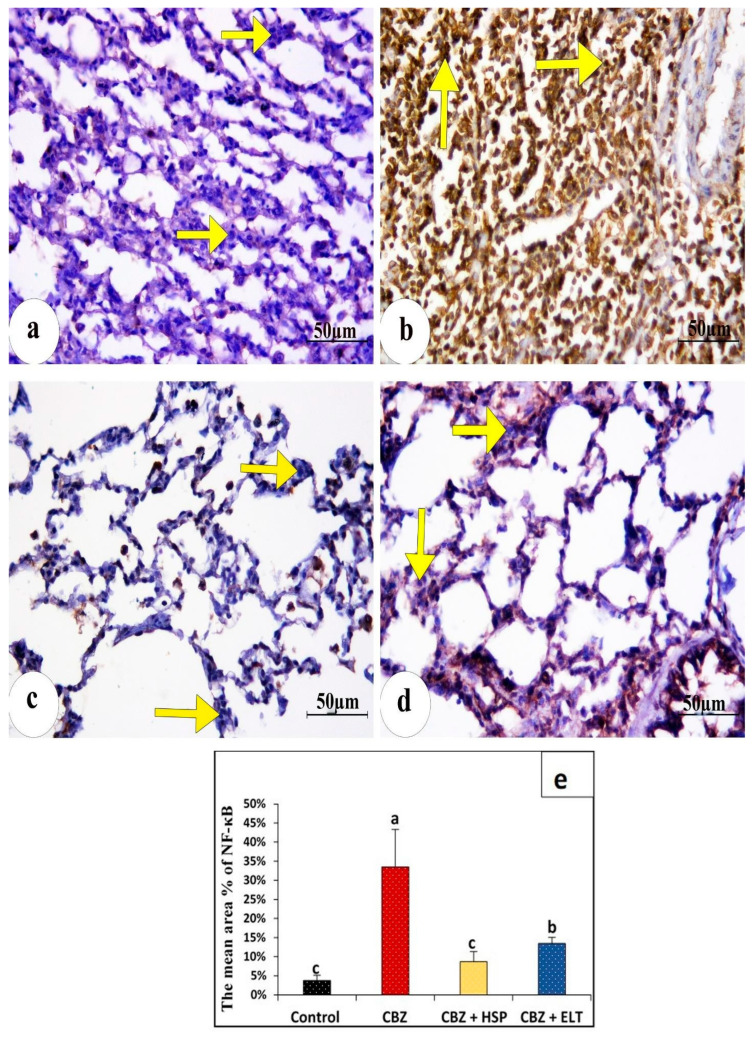
Photomicrographs of immunohistochemical expression of NF-κB in lung tissue in the different groups. (**a**) Control rats revealing very few expressions of NF-κB (arrows). (**b**). A significant increase in NF-κB expression (arrows) in the rats treated only with CBZ. (**c**) Expression of NF-κB (arrows) in the animals treated with HES plus CBZ was similar to the control rats. (**d**) Sections from rats treated with ELT plus CBZ illustrating a significant decrease in the overexpression of NF-κB (arrows). (NF-κB immunostaining, ×400). (**e**) Bar charts representation of the statistical analysis of the comparative quantification of the immunohistochemical expression for NF-κB in lung tissues of all. Sample number (*n*) = 6. Groups with the same superscript letter do not differ significantly. a: there is a significant difference compared with the control at *p* < 0.05. b, c: there is a significant difference compared with the CBZ group at *p* < 0.05.

**Figure 5:**
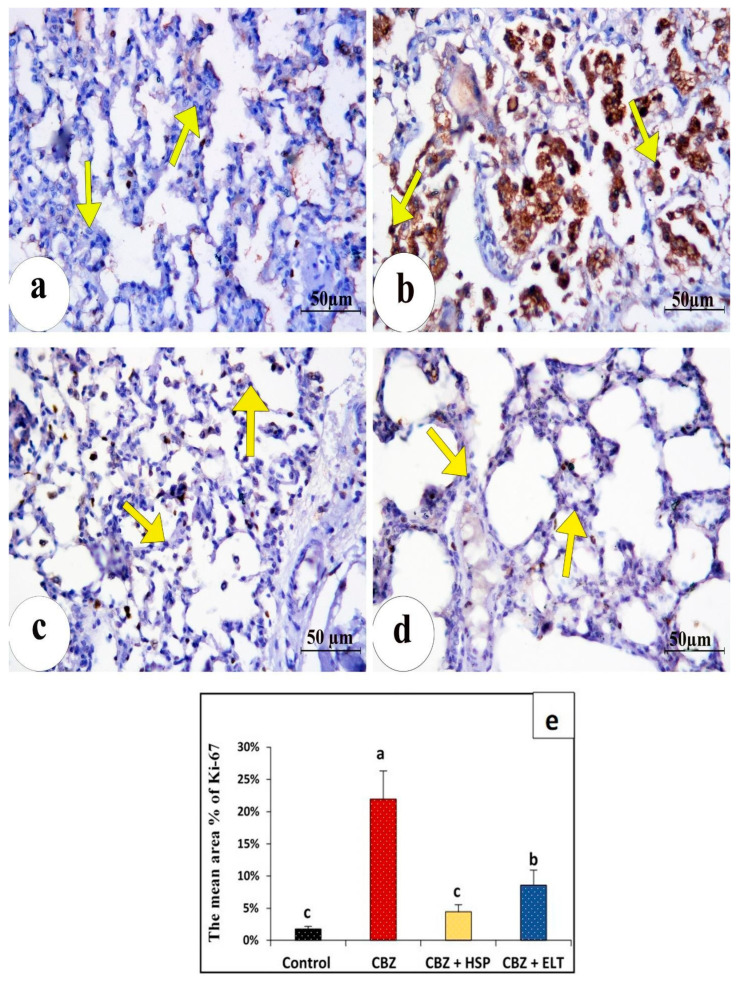
Immunohistochemical assessment of Ki-67 in lung sections of different groups. (**a**) the nuclei of group I (Control) illustrating minimal expression of Ki-67 (arrows). (**b**) The strongest Ki-67 expression intensity among the studied groups (arrows) in the nuclei of rats treated with only CBZ. (**c**) Moderate Ki-67 in the nuclei of the lung tissue of animals treated with HES plus CBZ (arrows). (**d**) mild Ki-67 expression in the nuclei of rats treated with ELT plus CBZ (arrows). (**e**) Bar charts representation of the statistical analysis of the comparative quantification of the immunohistochemical expression for Ki-67 in lung tissues of all groups. Sample number (*n*) = 6. Groups with the same superscript letter are not significantly different. a: there is a significant difference compared with the control at *p* < 0.05. b, c: there is a significant difference compared with the CBZ group at *p* < 0.05.

In the control group’s lung sections, a cytoplasmic iNOS immune reaction was found to be negatively reactive (Figure 3a). However, group II showed a strong immunopositive response (Figure 3b). Group III showed very low immunostaining (Figure 3c). Group IV showed moderate immunoreactivity (Figure 3d). In control lung sections, an NF-κB immunohistochemical analysis revealed no nuclear immunoreactions (Figure 4a). A significant positive dark brown nuclear reaction was visible in group II’s lung sections. (Figure 4b). In group III, the NF-B immunoreactivity was significantly decreased (Figure 4c). Lung sections of group IV illustrated weak nuclear immunoreactivity (Figure 4d). The Ki-67 nuclear immunoreaction was very weak in the control group (Figure 5a), while the CBZ-treated group expressed strongly positive nuclear immunoreaction (Figure 5b). The Ki-67 immunoreaction was low in group III (Figure 5c), but it was moderate in group IV (Figure 5d).

### 3.4. Morphometric Findings of iNOS, NF-κB, and Ki-67 Immunoreactivity

Statistical analysis of the comparative quantification of the immunohistochemical expression for iNOS, NF-κB, and Ki-67 in the lung tissue of rats from all groups is depicted by bar charts in Figure 3e, Figure 4e and Figure 5e. In the lung tissue of Group II, iNOS, NF- κB, and Ki-67 levels were significantly elevated as compared to the Control Group. Groups III and IV had significantly reduced levels of iNOS, NF- κB, and Ki-67 compared to Group II.

### 3.5. Ultrastructural Findings

**Figure 6:**
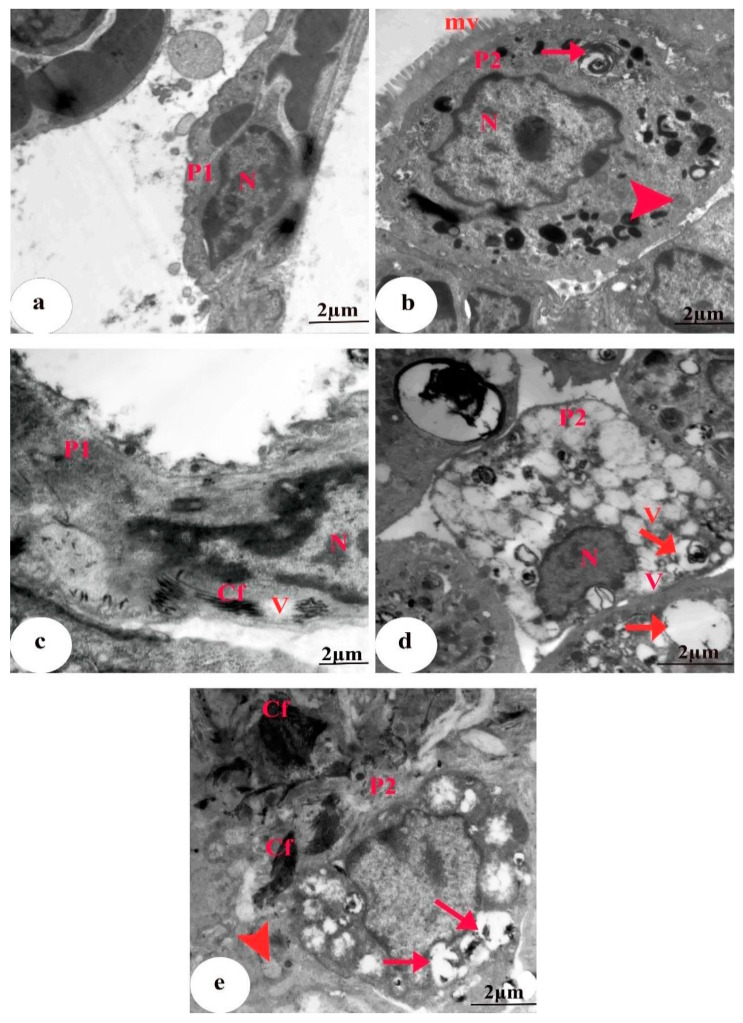
EM of a lung tissue section. (**a**,**b**) Control group revealing (**a**) type I pneumocytes (P1) with flat nucleus (N) surrounded by narrow perinuclear cytoplasm. Scale bar = 2 µm. (**b**) Type II pneumocyte (P2) cuboidal in shape with a large nucleus (N), lamellar bodies containing concentric secretory materials (surfactant) (arrow), mitochondria (arrowhead), and a few scattered microvilli (mv) on the surface. Scale bar = 2µm. Group II (**c**–**e**) rats treated with only CBZ showing (**c**) Type I (P1) containing nucleus (N) with heterochromatin clumps, deposition of collagen fibers (Cf), and some vacuoles (V). Scale bar = 2 µm. (**d**) degenerated Type II pneumocyte (P2) with an irregular shrunken pyknotic nucleus (N), lamellar bodies with a marked decrease of the surfactant material (arrows), absence of microvilli, and severe cytoplasmic vacuolation (V) were seen. Scale bar = 2 µm. (**e**) Type II pneumocyte (P2) containing numerous collagen fibers (Cf), empty lamellar bodies (arrows), and swollen vacuolated mitochondria with destroyed cristae (arrowhead.) were also detected. Scale bar = 2 µm.

**Figure 7:**
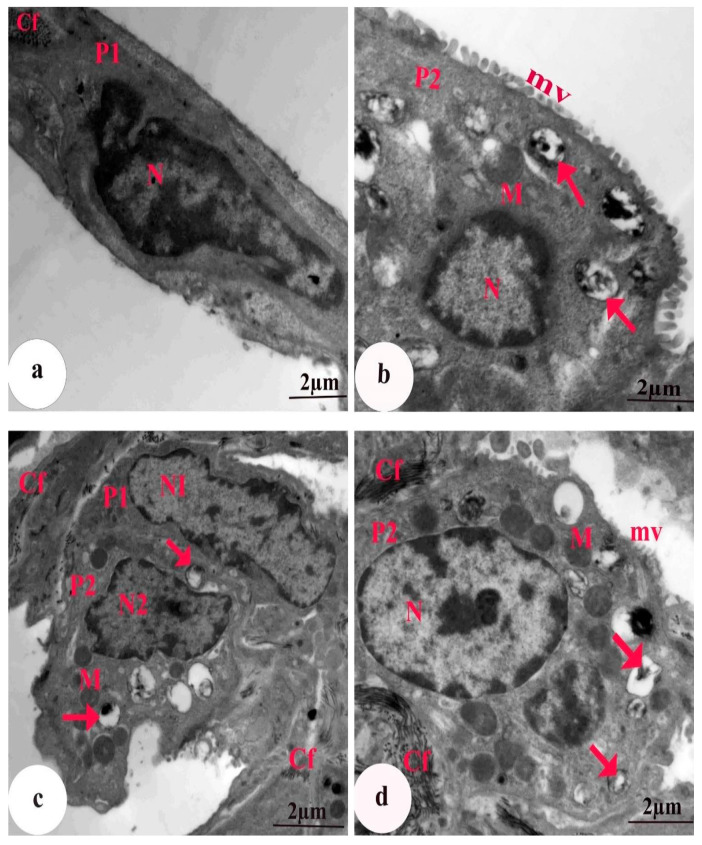
EM of a section of lung tissue. Group III (**a**,**b**) animals treated with CBZ plus HES showing (**a**) The alveolar type I cells (P1) appear nearly similar to the control, the nucleus (N) was elongated, and few collagen fibrils (Cf) were noticed. Scale bar = 2 µm. (**b**) type II pneumocyte (P2) contains a rounded nucleus (N) with peripheral heterochromatin, numerous lamellar bodies with a concentric lamella of secretory material (arrows), and intact microvilli (mv) were seen. Scale bar = 2 µm. Group IV (**c**,**d**) rats treated with ELT plus CBZ demonstrating (**c**) Pneumocyte type I (P1) with elongated nucleus (N1) and the adjacent pneumocyte type II (P2) containing a slightly normal nucleus (N2). Mitochondria (M), a moderate number of lamellar bodies (arrows), and few collagen fibers (Cf) were seen. Scale bar = 2 µm. (**d**) Mitochondria (M) in pneumocyte type II (P2), notice the nucleus (N) is elliptical, deposition of collagen fibrils (Cf) within the alveolar walls, some lamellar bodies were empty (arrows) and short surface microvilli (mv) were also observed. Scale bar = 2 µm.

The EM graph of a section of lung tissue from the control group revealed Type I alveolar cells with a flat nucleus within a narrow perinuclear cytoplasm. Type II alveolar cells were cuboidal with a large nucleus, lamellar bodies containing concentric secretory materials (surfactant), mitochondria, and a few scattered microvilli on the surface (Figure 6a,b). Group II rats treated with CBZ alone showed a Type I-containing nucleus with heterochromatin clumps, deposition of collagen fibers, and some vacuoles. Degenerated Type II pneumocytes with an irregular shrunken pyknotic nucleus, lamellar bodies with a marked depletion of the surfactant material, absence of microvilli, severe cytoplasmic vacuolation, numerous collagen fibers, and bulged vacuolated mitochondria with destroyed cristae were also detected (Figure 6c–e). Group III animals treated with HES + CBZ had alveolar Type I cells that appeared similar to those of the control, with an elongated nucleus and few collagen fibrils. Type II pneumocytes containing a rounded nucleus with peripheral heterochromatin, numerous lamellar bodies with a concentric lamella of secretory material, normal mitochondria, and intact microvilli were observed (Figure 7a,b). Group IV rats treated with ELT + CBZ had Type I pneumocytes with an elongated nucleus, and the adjacent Type II pneumocytes contained a slightly normal nucleus. Slightly normal mitochondria, a moderate number of lamellar bodies, few collagen fibers, and short microvilli on the surface were also observed (Figure 7c,d).

## 4. Discussion

The early identification and management of hyper or hypothyroidism require strict attention. Putting the inflammation and oxidative stress critical role in hypothyroidism into consideration, the antioxidant, anti-inflammatory, and anti-proliferative effects of HES are the main research focus. The present work posited that oxidative stress is the primary source of HPO-induced histopathological, immunohistochemical, ultrastructural, and biochemical abnormalities of the lung because antioxidant enzyme activity is suppressed, resulting in the formation of ROS and lung tissue injury. HPO may affect respiration by different mechanisms, even in subjects with unharmed respiratory systems. Insight into the systemic nature of chronic obstructive pulmonary disease (COPD) may be gained from understanding thyroid gland function and thyroid diseases. HPO increases the risk of COPD [47]. Clinical studies have proved the efficacy of HES and other flavonoids as antioxidants and their ability to defend against many chronic lung syndromes. According to Knekt et al. [48], people who consume a lot of dietary flavonoids may have a lower chance of developing chronic lung disorders. For all that, this research relied mainly on proving the antioxidant, anti-inflammatory, and anti-proliferative mechanisms of HES against CBZ-induced lung injury.

In the current study, the occurrence of HPO was confirmed in Group II (CBZ). Abdel-Wahhab and colleagues [49] reported a direct relation between excess TSH and increased oxidative stress. It is noteworthy that HES significantly increased FT3 and FT4 but ELT significantly increased FT3 while not significantly increasing FT4. These results agreed with those of Amra et al. [50], who found that ELT ameliorates the general toxic effects caused by CBZ-induced HPO, and Hegazy, et al. [51], who clarified that HES significantly boosts THs and decreases TSH when used in treatment with CBZ.

The formation of ROS is a major stress mechanism that causes oxidative stress in different organs, involving the lungs [52]. Additionally, since cellular injury brought on by ROS is the most hazardous effects of oxidative stress, it is useful to evaluate the oxidation product levels as indicators of such stress [53]. MDA formation is a toxicity marker of membrane injury and is a well-known secondary metabolites of lipid peroxidation [54]. In this study, the decreased GSH levels, CAT, and SOD activities in Group II may be attributed to ROS levels and lipid peroxidation levels. Nrf2 and HO-1 were significantly reduced in Group II compared to the control. In comparison to Group II, groups III and IV exhibited a significant augmentation in Nrf2 and HO-1. This suggests that CBZ had a pathophysiological effect on stress-provoked lung injury in the rat model. Inhibition of the master regulator of many antioxidants genes, Nrf2 renders cells more vulnerable to the harmful effects of ROS and pro-inflammatory motivators [55]. The current study hypothesized that HES has the ability to activate antioxidant enzyme production by stimulating Nrf2/HO-1 signaling pathway.

In this work, IL-35 levels in Group II significantly decreased, whereas IL-35 levels in Groups III and IV significantly rose. These results confirm the immunomodulatory effect of HES against lung inflammation. The results of Group II in this study demonstrated a remarkable increase in IL-6 and TNF-α. This could be explained by Kandhare et al. [56], who clarified that the exacerbated input of ROS due to pulmonary insult results in the liberation of the pro-inflammatory cytokines TNF-α and ILs during the primary stages of the disease. Inflammation provokes oxidative stress by generating ROS at the site of inflammation, which exaggerates inflammation by activating various pathways, particularly transcription factor NF-κB. [57]. Many diseases, such as chronic inflammation, tumors, and autoimmune diseases, may occur as a result of dysregulated NF-κB activation. That’s why NF-κB is firmly regulated to maintain homeostasis. The pro-inflammatory cytokine IL-6 is released from endothelial cells, monocytes, B lymphocytes, and fibroblasts and promotes antibody production and lymphocyte differentiation [58]. By upregulating IL-10 secretion and decreasing the expression of pro-inflammatory cytokines TNF-α, IL-6, and -17, the anti-inflammatory and anti-apoptotic IL-35 recombinant protein reduces inflammation and reverses the inflammatory response. [59]. These findings are in strong agreement with Yeh et al. [60] who clarified the immunomodulation of HES against endotoxin-induced acute pulmonary damage.

The oxidative stress marker iNOS, which is a type of nitric oxide (NO) synthase, is directly linked to the immune system and produces NO [61]. Large quantities of NO produced by iNOS may be responsible for the damage seen in various experimental models of inflammation (Antosova et al. 2017). NF-κB regulates the transcription of a variety of cytokine genes, involving interleukin genes and TNF-α when it is active, moving from the cytoplasm to the nucleus of the cell [62]. Ki-67, a nuclear antigen found in proliferating cells, is the furthermost extensively utilized proliferation-linked marker [63]. The current study agrees with Wang et al. [64] who manifested the role of HES in reducing oxidative stress and inflammatory responses in lung tissues. By blocking the NF-κB signaling pathway, HES controlled the levels of inflammatory factors and oxidation/antioxidant indicators. These findings agreed with Hristova et al. [65] and Yang et al. [66] who confirmed suppressed pro-inflammatory cytokines and iNOS expression by the upregulated HO-1 pathway.

In the current study, the biochemical and immunohistochemical results were synchronized with the histopathological and ultrastructural results. The cytoarchitecture, cellular discontinuity, absence of cilia, cellular atrophy of the alveoli, a marked decrease of the surfactant, bulged vacuolated mitochondria, and epithelial lining of the bronchioles was detected. Histologically, methimazole induced HPO, worsening lung cells. Cano-Europa et al. [67] suggested that THs deficiency lowers the activity of Na^+^–K^+^–ATPase, alters apical Na^+^ conductance, and increases extracellular fluid, causing alveolar injury. Lipid peroxidation is linked to several negative consequences, including exacerbated membrane stiffness and decreased mitochondrial lifespan [68,69].

Alveolar collapse is provoked in this study by the promotion of lipid peroxidation in Type II pneumocytes, which results in decreased surfactant synthesis and the resultant alveolar collapse. These outcomes are consistent with those of Mirastschijski et al. [70], who confirmed that lung surfactant reduces surface tension, preventing alveolar collapse during exhalation. In addition, ROS activation alters the antioxidant systems and increases lipid peroxidation-caused degenerative alterations in Type II pneumocytes [71].

The present study also found that CBZ treatment provoked an increase in inflammatory mononuclear cells in the peribronchial and perivascular areas. Oxidative stress-sensitive signaling pathways provoked vascular congestion and cellular infiltration, which is caused by the destruction of the endothelium barrier, affecting vascular integrity and promoting permeability, and resulting in inflammation [72]. Inflammation in the lungs is marked by changes in vascular thickness, leukocyte infiltration, and an increase in macrophages, neutrophils, and lymphocytes. Worsened pulmonary cells, according to Robb et al. [73], emit higher levels of inflammatory cytokines, prompting autoimmune reactions and hypersensitivity. Furthermore, red blood cell extravasation might cause inflammation by drawing mononuclear cells to the congestion site. Many types of cells, including immune cells, inflammatory cells, and fibroblasts, move to regions of injury following tissue damage, in addition, numerous cytokines are released, which add a new load of cell inflammation [74].

In this study, we observed that CBZ treatment caused hemorrhage, alteration of the epithelial lining of the bronchiole, and thickened and increased connective tissue in the alveolar septa. The thickened interalveolar septa, according to Larsen et al. [75], were probably caused by nonspecific interstitial pneumonitis, which is mostly comprised of mononuclear cells. Pro-inflammatory cytokines cause oxidative stress, leading to an increase in the production of ROS, which has been linked to pulmonary secondary endothelial injury [76].

This study revealed fatty cellular infiltration, which is a common hallmark of the development of lung disease. Our data added to the validity of some theories, and these findings were consistent with those of El Ayed et al. [77], who demonstrated that lipid accumulation in the lung causes oxidative stress via increased levels of free radicals. There is enhanced lung surfactant lipids oxidation during inflammatory reactions, which can be harmful if they accumulate. Lung damage is caused by oxidized lipid-induced cytotoxicity, which stimulates the inflammatory response [78].

Our findings on the deposition of collagen fibers in group II and marked hyperplasia of the bronchioles with a significant increase in Antigen Ki-67 in the lung tissues were congruent with those of Wang et al. [79], who posited that the constant influx of pro-inflammatory cytokines results in a persistent state of unrestricted inflammation, proliferation, and fibrosis, limiting lung tissue repair. TNF-α, a strong pro-inflammatory cytokine, regulates the advancement of the fibrotic process through a complex network of cellular and molecular interactions [80]. In the lungs, the NF-κB signaling pathway is activated, resulting in increased pulmonary alveolar well thickness and fibrotic changes in the tissues [81]. increased TSH levels in this study were linked to an increase in fibrosis risk [82]. Fibrosis of the lung can be promoted by HPO and can be upturned by the administration of THs [22].

HES could significantly reduce inflammation, fibrosis, the number of inflammatory cells, pulmonary edema, alveolar thickness, and thrombosis [83]. Interestingly, the ultrastructural and histopathological alterations in the lung tissues were ameliorated by HES in Group III and ELT in Group IV. Additionally, HES and ELT reduced lung leukocyte buildup and collagen deposition while preserving the alveolocapillary’s structural integrity. Thus, HES may afford a natural substitutive therapeutic for the controlling of HPO and an adjunct to enhance the side impacts caused by CBZ. This study agrees with Zhou et al. [84] who manifested that the lung tissues section of HES (100 mg/kg) treated rat revealed the existence of few inflammatory cells, the normal structure of type II alveolar cells and bronchial epithelial cells, and a distended vesicle with cytoplasmic vesicular granules. There are some limitations to the current study, so further research is needed to figure out the pathophysiological connections between HPO and lung injury and exactly how the HES protection mechanism works. In addition, the study needed the determination of fibrotic markers and further proliferative markers other than Ki-67.

## 5. Conclusions

Overall, our biochemical, histological, immunohistochemical, and ultrastructural findings suggest that CBZ causes harmful effects on rats’ lungs. These findings indicate that concomitant HES at a high dose (200 mg/kg) with CBZ effectively protects the lung tissue from the severe damage provoked by CBZ by acting on different levels. The potential effect of HES is mediated through the improvement of THs, the suppression of NF-κB pathways which improve the modulation of oxide-inflammatory markers (Nrf2 and HO-1), inhibition of the pro-inflammatory markers (TNF-α, and IL-6), increasing of anti-inflammatory marker IL-35 and decreasing of ki-67 to diminish pulmonary injury. The present study confirmed that HES is a strong antioxidant and anti-inflammatory in the lung of the CBZ model (Figure S1).

## Figures and Tables

**Table 1:** The efficacy of HES in comparison with ELT on CBZ-induced alteration in TSH, FT3, FT4, IL-35, TNF-α, and IL-6 in lung tissues of the study groups.

Groups	TSH (μiu/ml)	FT3 (pg/mL)	FT4 (ng/dl)	IL-35 (pg/mg)	IL-6 (pg/mg)	TNF-α (pg/mg)
Group 1 (Control)	0.008 ± 0.00342 ^b^	5.10 ± 0.462 ^b^	3.41 ± 0.129 ^c^	308.1 ± 23.9 ^c^	65.4 ± 2.7 ^c^	13.8 ± 0.6 ^c^
Group II (CBZ)	0.011 ± 0.000760 ^a^	2.96 ± 0.246 ^a^	2.50 ± 0.162 ^a^	120.5 ± 1.4 ^a^	205.8 ± 5.3 ^a^	81.7 ± 2.7 ^a^
Group III (HES + CBZ)	0.007 ± 0.000258 ^b^	4.34 ± 0.327 ^b^	2.95 ± 0.113 ^b^	169.0 ± 9.7 ^b^	109.3 ± 4.8 ^b^	26.1 ± 1.6 ^b^
Group IV (ELT + CBZ)	0.006 ± 0.000654 ^b^	4.30 ± 0.251 ^b^	2.75 ± 0.147 ^ab^	172.8 ± 8.5 ^b^	107.9 ± 4.3 ^b^	26.4 ± 2.3 ^b^

This test was repeated two times. Sample number (n) = 6. Values are represented as Mean ± standard error (SE). Different superscript letters represent significantly different (*p* < 0.05) values. ^a^: there is a significant difference compared with the control at *p* < 0.05. ^b,c^: there is a significant difference compared with the CBZ group at *p* < 0.05. Abbreviations: HES: hesperidin; ELT: Eltroxin; CBZ: carbimazole; TSH: thyroid-stimulating hormone; FT3: Free triiodothyronine; FT4: free thyroxine; IL: interleukin; TNF-α: tumor necrosis factor-alpha.

## Data Availability

The authors confirmed that All data generated or analyzed during this study are included in this published article.

## Supplementary Materials

The following supporting information can be downloaded at: https://www.mdpi.com/article/10.3390/biomedicines11061570/s1. Figure S1: A summary of the probable protective mechanisms of hesperidin on lung; Table S1: Tests of Normality.
