# Supplementary figures and images for: Hesperidin Attenuates Hypothyroidism-Induced Lung Damage in Adult Albino Rats by Modulating Oxidative Stress, Nuclear Factor Kappa-B Pathway, Proliferating Cell Nuclear Antigen and Inflammatory Cytokines
Source: Biomedicines. 2023 May 29;11(6):1570. doi: 10.3390/biomedicines11061570 (PMC10295994; doi:10.3390/biomedicines11061570)

**
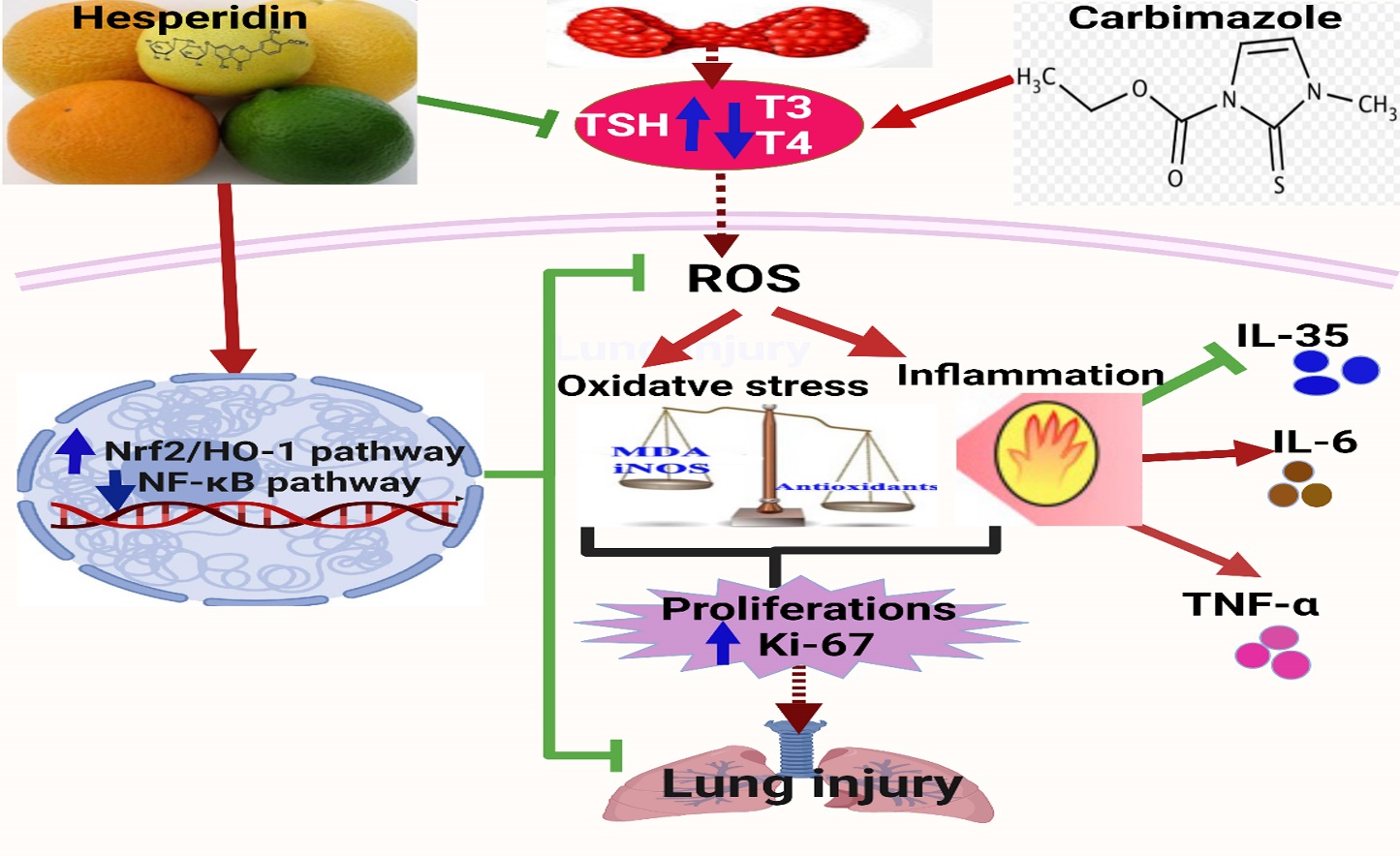
**

**Figure S1:** A summary of the probable protective mechanisms of hesperidin on lung.

Supplement: Supplementary file 1 [file biomedicines-11-01570-s001.zip › supplmentary fig. S1.docx]
